# HPLC-DAD-MS Characterization, Antioxidant Activity, α-amylase Inhibition, Molecular Docking, and ADMET of Flavonoids from Fenugreek Seeds

**DOI:** 10.3390/molecules28237798

**Published:** 2023-11-27

**Authors:** Mohammed Lamine Khenifi, Talia Serseg, Piotr Migas, Mirosława Krauze-Baranowska, Sadin Özdemir, Chawki Bensouici, Mohammed I. Alghonaim, Khattab Al-Khafaji, Sulaiman A. Alsalamah, Messaoud Boudjeniba, Mohamed Yousfi, Fehmi Boufahja, Hamdi Bendif, Mohamed Mahdid

**Affiliations:** 1 Laboratoire d’Ethnobotanique et Substances Naturelles, Departement of Biology, Ecole Normale Supérieure de Kouba, B.P 92, Kouba 16308, Algeria; m.khenifi@ens-lagh.dz (M.L.K.); mboudjeniba@yahoo.fr (M.B.); hamdi.bendif@univ-msila.dz (H.B.); mahdid_m@yahoo.fr (M.M.); 2 Laboratoire des Sciences Appliquées et Didactiques, Ecole Normale Supérieure de Laghouat, B.P 4033 la gare routière, Laghouat 03000, Algeria; t.serseg@lagh-univ.dz; 3 Fundamental Sciences Laboratory, Amar Telidji University, Laghouat 03000, Algeria; m.yousfi@lagh-univ.dz; 4 Pharmacognosy with Medicinal Plants Garden, Medical University of Gdańsk, 80-416 Gdańsk, Poland; pmig@gumed.edu.pl (P.M.); krauze@gumed.edu.pl (M.K.-B.); 5 Food Processing Programme, Technical Science Vocational School Mersin University, Mersin TR-33343, Turkey; sadinozdemir@mersin.edu.tr; 6 Biotechnology Research Center (CRBT), Ali Mendjli New Town UV 03, B.P E73, Constantine 25016, Algeria; c.bensouici@crbt.dz; 7 Biology Department, College of Science, Imam Mohammad Ibn Saud Islamic University (IMSIU), Riyadh 11623, Saudi Arabia; mialghonaim@imamu.edu.sa (M.I.A.); saalsalamah@imamu.edu.sa (S.A.A.); 8 Department of Environmental Science, College of Energy and Environmental Science, Al-Karkh University of Science, Baghdad 10081, Iraq; k.a.alkhafaji@gmail.com; 9 Department of Natural and life Sciences, Faculty of Sciences, University of Msila, Msila 28000, Algeria

**Keywords:** Fenugreek, *Trigonella foenum-graecum*, HPLC-DAD-ESIMS, flavonoid glycosides, α-amylase, antioxidant activity, molecular docking, molecular dynamic simulation

## Abstract

Fenugreek (*Trigonella foenum-graecum*) has a great beneficial health effect; it has been used in traditional medicine by many cultures. Likewise, the α-amylase inhibitors are potential compounds in the development of drugs for the treatment of diabetes. The beneficial health effects of fenugreek lead us to explore the chemical composition of the seeds and their antioxidant and α-amylase inhibition activities. The flavonoid extraction from fenugreek seeds was achieved with methanol through a Soxhlet apparatus. Then, the flavonoid glycosides were characterized using HPLC-DAD-ESI-MS analysis. The antioxidant capacity of fenugreek seed was measured using DPPH, FRAP, ABTS, and CUPRAC assays. Finally, the α-amylase inhibition activity was carried out using in vitro and in silico methods. The methanolic extract was found to contain high amounts of total phenolics (154.68 ± 1.50 μg GAE/mg E), flavonoids (37.69 ± 0.73 μg QE/mg E). The highest radical-scavenging ability was recorded for the methanolic extract against DPPH (IC_50_ = 556.6 ± 9.87 μg/mL), ABTS (IC_50_ = 593.62 ± 9.35 μg/mL). The ME had the best reducing power according to the CUPRAC (A 0.5 = 451.90 ± 9.07 μg/mL). The results indicate that the methanolic extracts of fenugreek seed best α-amylase inhibition activities IC_50_ = 653.52 ± 3.24 μg/mL. Twenty-seven flavonoids were detected, and all studied flavonoids selected have good affinity and stabilize very well in the pocket of α-amylase. The interactions between the studied flavonoids with α-amylase were investigated. The flavonoids from fenugreek seed present a good inhibitory effect against α-amylase, which is beneficial for the prevention of diabetes and its complications.

## 1. Introduction

Fenugreek (*Trigonella foenum-graecum* L.) is an herbaceous aromatic leguminous [1] used for culinary and medicinal purposes [2]. It is largely cultivated all over the world [3]. It has been found to be rich in bioactive secondary metabolites such as phenolics, flavonoids, alkaloids, saponins, carbohydrates, vitamins, volatile oils [4,5], triterpenoids [6], and coumarin [1]. Previously reported that major chemical constituents of fenugreek are steroidal sapogenins and nicotinic acid [1,3,4], trigocoumarin, diosgenin, trimethyl coumarin, and trigonelline [7,8,9,10,11], fenugreekine, scopoletin, and phytic acid [12]. Fenugreek has been used widely in traditional medicine due to its medicinal properties. Traditionally used in Ayurvedic and Chinese medicine as a remedy against diabetes, constipation, anemia, fever, and stomach disorders [2,4]. It has been reported that fenugreek is a powerful antioxidant source [13]. It has also been described to exhibit pharmacological effects such as antidiabetic, antimicrobial, hypocholesterolemic, anti-inflammatory, antipyretic, chemopreventive, anticancer, gastroprotective, hepatoprotective, antioxidant, appetite stimulation, and good lactating aid in weaning mother [14,15,16].

Diabetes mellitus is a metabolic disorder characterized by chronic hyperglycemia as a result of a deficiency in insulin secretion or its action or both [17,18,19,20,21], which leads to disturbances in carbohydrate, fat, and protein metabolism [19]. According to WHO, in 2014, there were 422 million adults living with diabetes worldwide. Diabetes mellitus (DM) accounted for 6.7 million deaths globally in 2021, with expenditures of USD 966 billion. Mortality is predicted to rise nearly 10-fold by 2030 [22] to a projected 783 million in 2045 [23]. The rise of glucose levels in the blood is a result of carbohydrate hydrolysis, a process primarily catalyzed by the enzymes α-glucosidase and α-amylase [24]. The process involves the hydrolysis of carbohydrates by the salivary α-amylase enzyme to disaccharides and oligosaccharides, which are further hydrolyzed by the α-glycosidase enzyme to monosaccharides like glucose, which are absorbed into the blood through the small intestine [25]. The pancreatic α-amylase then hydrolyzes the remaining oligosaccharides to glucose and maltose [26,27,28]. So, α-amylase is a key enzyme for starch digestion [29]. Inhibition of α-amylase can help in reducing hyperglycemia [30]. Pharmacologically, α-amylase inhibitors may be employed for the decrease in glucose absorption rate and, subsequently, reduce the postprandial rise in plasma glucose and the risk for long-term diabetes complications [31]. Currently, α-amylase inhibitors such as acarbose are introduced to the market as a treatment for diabetes [26,32]. The current anti-diabetes drugs can cause side effects such as current anti-diabetes drugs can cause side effects such as decreased appetite, diarrhea, stomach pain, and extreme tiredness, which reduce patient compliance and treatment effectiveness [33]; therefore, efforts to find natural inhibitory components that could replace these medications have gained more attention [34]. Flavonoids are a group of compounds that can inhibit amylase enzyme activity [35].

Moreover, molecular docking has been frequently used in drug design, aiming to predict binding sites of a ligand with a target protein. The result of a ligand on the target protein could be predicted by comparing its binding site with an established drug (e.g., inhibitor), which has known action on that protein. Likewise, molecular docking has been applied to identify key active compounds that can bind to the corresponding targets at the known active site. To the best of our knowledge, the inhibitory effect of compounds from fenugreek against α-amylase was investigated for the first time using in silico methods in this study.

Oxidative stress is a common cause of many diseases, like diabetes, high blood pressure, preeclampsia, atherosclerosis, acute renal failure, Alzheimer’s, and Parkinson’s [36,37,38,39]. It results from the loss of balance between oxidation and antioxidation processes, increasing free radicals in the body and leading to deficient cell operation [40,41], causing cell damage and dysfunction [42]. Several studies demonstrate that antioxidants are important to maintain body health due to their capacity to neutralize free radicals, leading to a decrease in oxidative stress [43]. The antioxidants can be found naturally in plants, microorganisms, and animals or chemically synthesized [43,44]. The possible toxicological risks of synthetic antioxidants have been reconsidered, leading researchers to seek natural antioxidants that have more benefits and are less toxic [45,46,47]. Natural antioxidants have gained significant attention due to their potential to improve antidiabetic therapy [48] and may reduce or prevent Type II diabetes through a number of processes, including (1) lowering mitochondrial oxidative stress, (2) decreasing the adverse effects of lipid peroxidation, and (3) contributing as important cofactors for antioxidant enzymes [49].

In this study, we aim to explore the chemical composition of crude extract of fenugreek seeds using HPLC-DAD-ESIMS analysis and investigate the antioxidant and antidiabetic effects with the aid of computational approaches followed by in vitro experiments.

## 2. Results and Discussion

### 2.1. HPLC-DAD-ESIMS Analysis

**Figure 1:**
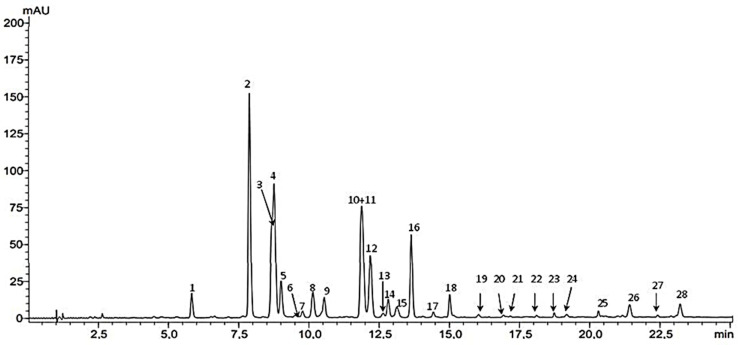
One-dimensional HPLC chromatogram of the methanol extract from fenugreek seeds. Kinetex PFP column, gradient elution; detection UV at λ 330 nm.

Based on literature data [4,50,51], the separation of flavone C-glycosides from fenugreek seeds was carried out mainly on HPLC columns, where the stationary phase was C-18 silica gel, but for the first time, a Kinetex PFP column (100 × 46 mm, 2.6 µm) was chosen for HPLC analysis of these compounds. The gradient elution program was optimized and included increasing the concentration of the water/trifluoroacetic acid mixture 100:0.1 (*v*/*v*) from 10% to 50% in the acetonitrile/trifluoroacetic acid mixture 100:0.1 (*v*/*v*) (tG 30 min). The separation conditions used enabled the identification of all flavonoid compounds present in the tested plant material. In the analyzed methanol extract, twenty-seven flavonoids were identified “See Figure 1 below” (compounds **1**–**23**, **25**–**28**) (Table 1), including twenty-two flavones (**1**–**5**, **7**–**12**, **14**–**19**, **23**–**25**, **26**–**28**) and three flavonols (**6**, **13**, **21**). The presence of ten compounds was confirmed by comparing their chromatographic data: tR and UV and MS values with standard compounds, and these components of the extract were identified as: vicenin 2 (1), vicenin 1 (**2**), schaftoside 2 (**3**), schaftoside 1 (**4**), isoorientin (**5**), orientin (**8**), vicenin 3 (**9**), isovitexin (**11**), vitexin (**13**) and quercetin 3-O-rhamnoside (**21**). All flavone C-glycosides (**1**–**5**, **8**–**9**, **11**, **13**) identified by us have been previously described in *Trigonella foenum-graecum* seeds [50,51]. On the other hand, the presence of quercetin 3-O-glycosides was previously shown only in fenugreek herb [52]. Other flavonoids were identified on the basis of the obtained UV and ESI-MS spectra (Table 1) and their comparison with literature data [50,51,52,53,54]. Eight compounds (**7**, **10**, **14**–**19**) were assigned to flavone–apigenin derivatives based on the presence of two absorption maxima in the UV spectra at λmax 335–339 nm (band I) and at λmax 269–271 nm (band II) [54]. Of these, six compounds (**10**, **14**–**18**) have been tentatively identified as apigenin-C-di-(6/8)-pentoside isomers based on molecular ion values at *m*/*z* 535 [M + H]+ and *m*/*z* 533 [M − H]^−^ and its adduct with TFA at m/z 647 [M − H + TFA]^−^ [4,50,51]. On the other hand, two other apigenin derivatives (**7**, **19**) were initially identified as an isomer of apigenin C-di-6,8-pentoside-hexoside (**7**) [50] and, as a new in fenugreek, apigenin C-di-(6,8)-pentoside methyl ether (**19**), taking into account in their MS spectra the values of molecular ions and its adducts with TFA, respectively for seven at *m*/*z* 565 [M + H]^+^/563, 677 [M − H]^−^, [M − H +TFA]^−^ and for 19 at *m*/*z* 549 [M + H]^+^/547, 661 [M − H]^−^, [M −H + TFA]^−^. In the tested group of flavonoid compounds of fenugreek seeds, the occurrence of ester derivatives of flavones (**26**–**28**) and flavonols (**6**, **13**) was also demonstrated (Table 1). The presence of the latter was revealed in this plant’s raw material for the first time. Analyzing the band II absorption maxima in the UV spectra of **6** and **13**, compound **6** was identified as a kaempferol derivative (λmax 268 nm) and compound **13** as a quercetin derivative (λmax at 253 nm with a shoulder (s) at 265 nm, which confirms the presence of an o-dihydroxyl group in the side phenyl) [54]. In addition, shifts of the absorption maxima of band I towards low wavelengths [for six λmax 324 nm and thirteen λmax 315, 349 (s)] were observed, confirming the fact that both compounds are ester derivatives [50,51,54]. Both compounds have been tentatively identified as kaempferol O-feruloyl-triglucoside/trigalactoside isomer (**6**) and quercetin-O-coumaroyl-triglucoside/trigalactoside isomer (**13**) basing on their MS spectra and the values of molecular ions and its adducts with TFA, respectively for six at *m*/*z* 949 [M + H]^+^/947, 1061 [M − H]^−^, [M − H +TFA]^−^ and for 13 at *m*/*z* 935 [M + H]^+^/933, 1047 [M − H]^−^, [M − H + TFA]^−^. Flavonols, derivatives of kaempferol and quercetin, including triglycosides, as well as their esters, are the main components of the aerial parts of fenugreek [52]. In the tested *Trigonella foenum-graecum* seeds, the presence of three other flavone glycoside esters was confirmed by HPLC-DAD-MS, namely p-coumaryl derivatives of orientin/isoorientin (**26**) and p-coumaryl derivatives of vitexin/isovitexin (**27**,**28**) [50,51].

### 2.2. TPC, TFC, and Antioxidant Activity

The pharmacological effects of fenugreek seeds may be a result of the several secondary metabolites presence, such as phenols and flavonoids. The extraction yield, total phenolics, and flavonoid content in the methanolic extract from fenugreek seeds obtained by Soxhlet are shown in Table 2.

Secondary metabolites and composites extracted from plants have shown a beneficial impact on an individual’s health, such as flavonoids and phenolic compounds. Thus, our study shed light on the fenugreek, where the yield of extraction for fenugreek seeds was 17.6%, using 10 g of seed powder (Table 2), which is consistent with [55] findings that reported a yield portion of 17.66%, using 10 g of fenugreek seeds, whereas [2] findings had shown much more yield, in which that latter is about 20.25% while the value ranged between 9.68% and 25.89%, using multiple organic solvents [56]. Therefore, the yield of extraction can be influenced by many factors, such as genetic factors, environment, extraction manner, solvent type, and its polarity, as well as the ratio of the solvents with the plant matter [57,58,59].

Polyphenols are secondary metabolites naturally produced by plants [60] to be protected from biotic and abiotic stress [61]. They have anti-inflammatory, antioxidant, and antimicrobial activities and anti-coronavirus properties [62]. Table 2 illustrates the findings of the measured phenolic and flavonoid content in the methanol extract, in which the phenolic content was 154.68 ± 1.50 µg of GAE/mg of the extract. Meanwhile, the total flavonoid compounds were 37.69 ± 0.73 µg of QE/mg of the extract. Thus, the obtained findings were the best according to [63] results, in which they figured out that the total content of polyphenols and flavonoids of the ethanol extract was 9.7 mg of GAE/g of the extract, and 14.6 mg of QE/g of the extract respectively; hence the phenolic content is affected by the solvent and its polarity [64]. Also, according to [65], methanol extract contains more phenolic and flavonoids than ethanol extract because methanol is more polar than ethanol. This divergence is also due to several factors, including type, environmental factors, and agriculture techniques [66].

Oxidation stress is associated with many diseases, such as diabetes, atherosclerosis, hypertension, respiratory diseases, arthritis, cataracts, cancer, cardiovascular disease, etc., that are caused by free radicals [67]. The currently used antioxidants, such as BHA, BHT, PG, and TBHQ, are responsible for liver damage and cancer [68].

There is great interest in developing new antioxidants used in the pharmaceutical, cosmetic, and food industries. Also, botanical extracts with antioxidant capacity may work through various mechanisms, including oxygen root absorption, energy reduction, and free radical removal [69]. Therefore, it is crucial to use several methods based on multiple chemical reactions to determine antioxidant activity [70]. Accordingly, to examine methanolic extract antioxidant activity, four tests have been performed, namely, DPPH, ABTS, FRAP, and CUPRAC, and the findings are shown in Table 3.

The DPPH and ABTS tests were used to measure the extract radical scavenging, where there was an equinox in the activity, which indicated an IC_50_ equal to IC_50_ = 556.6 ± 9.87 µg /mL and 593.62 ± 9.35 µg /mL, respectively. This result is stronger than that found by [71], in which IC_50_ values varied between 80 and 149 mg/mL of the methanol extract of four fenugreek cultivars, But compared our finding with those of [72] with 172.6 ± 3.1 µg/mL in fenugreek seed oil was with DDPH test, our results still weak while, we had obtained better results with ABTS test, than those of [73], who stated that the IC_50_ value was 962.5 µg /mL of the fenugreek seeds ethanol extract. While compared with [72], who found the antioxidant capacity of the oil by ABTS assay (IC_50_ = 161.3 ± 2.21 µg/mL), our findings are weak. This is because antioxidant activity compounds dissolve in methanol better than ethanol because of methyl radical presence that is shorter than the ethyl radical present in ethanol, which leads to more solvation of the antioxidant molecules [74].

Besides, the antioxidant activity was also examined using FRAP and CUPRAC to assess reduction capability. On one hand, the value, using CUPRAC test, was A0.50 = 451.90 ± 9.07 µg /mL of methanol extract, while it was 108.33 mg TE/g extract of fenugreek seeds [75], the value that does not reach A0.50, using iron reduction test, in which the maximum focus of the test was 200 µg/mL, and the samples did not show up to 50% of the reduction capacity within this range; hence, its value “A0.50” was reported to be greater than 200 µg/mL. The antioxidant activity and the reduction capacity of fenugreek seeds methanol extract are owing to the presence of phenolic and flavonoid compounds. According to [76], fenugreek can have an antioxidant ability because there is flavone C-glycoside.

Similarly, the antioxidant impact is caused by the presence of flavonoid compounds, such as rutin, apigenin 7-glucoside, Quercetin [77], kaempferol [78], as well as the antioxidant activity per compound is all about many composition factors, namely: Phenols Number; Hydroxy groups or Methoxy groups; and other compositions [79].

### 2.3. Anti-α-amylase Activity

Diabetes, also known as diabetes mellitus, is a chronic disease caused by a group of metabolic disorders characterized by a high blood glucose level (hyperglycemia) [80] as a result of the lack of insulin production or its ineffectiveness. There are only two treatments for this disease, which are insulin injections or long-term intake of antidiabetic drugs [81]. Therefore, α-amylase enzyme inhibitors are used to treat diabetes because they slow down the digestion of complex carbohydrates; consequently, glucose level gets low after eating [82]. On the one hand, the α-amylase enzyme can be found at the level of both saliva and pancreas because it is responsible for dividing long-chain carbohydrates (starch) and converting them into maltose. It is the α-glucosidase pillar in the small intestine that facilitates its absorption, leading to hyperglycemia (high blood glucose) [83]. On the other hand, In the case of diabetics, these enzyme inhibitors delay the complex carbohydrates division that leads to Diabetes hypoglycemia (a low level of glucose in the blood) after eating. [84]; thus, the α-amylase enzyme is the most important element that is needed in the digesting process of carbohydrates. Consequently, the inhibition of this enzyme leads to the inhibition of the digestion process of carbohydrates, resulting in diabetic hypoglycemia after eating [85].

Therefore, the current studies on anti-diabetic drugs have shown that botanical medicine has a great role in detecting high-potential plants to inhibit α-amylase enzymes. After all, an investigation on the anti-diabetes effect of fenugreek seed extract was carried out by assessing the methanol extract’s potential to inhibit the amylase enzyme. This approach aims to prevent hypoglycemia after eating by interfering with the decomposition and absorption of carbohydrates in the intestine [86], and the anti-diabetes effect of T *Foenum*-*graecum* extract (Reported using IC_50_ values) is illustrated in Table 2.

In addition, Methanol extract strongly inhibited the amylase enzyme compared to the positive control of acarbose, where for methanol extract, IC_50_ was valued at 653.52 ± 3.24 µg/mL, while the acarbose recorded value was 3650.93 ± 10.70 µg/mL.

However, [87] findings were found to be in approximate agreement with our findings, in which the IC_50_ value was 690 ± 0.01 µg/mL, whereas [88] did not notice any effect of methanol extract on the concentration of 2.5 mg/mL, in which IC_50_ value used to inhibit α-amylase of aqueous, hydroalcohol, and ethanol extracts 6.476, 12.395, and 8.690 mg/mL, respectively [89]. Also, the inhibition ratio in the concentration of 1 mg/mL was 37.7% [90], whereas in the [91] research study, the α-amylase inhibition ratio was 41.64%. In contrast with the study that was conducted by [92], which presented that there are relatively low molecular weight compounds in the water extract of fenugreek that inhibit α-amylase and Sucrase in rat intestines.

Finally, flavonoid-rich and phenols-rich extracts can contribute to diabetes management and complications [70,89]. Also, phenols play a great role in inhibiting α-amylase and inhibiting the absorption of glucose in the intestine. [93,94,95,96,97]; consequently, it has been reported that flavonoids are the active biological principle of most hypoglycaemic and anti-diabetic medicinal plants [98,99], and because there are flavone C-glycosides compounds, digestive enzymes were inhibited [100,101]. Also, fenugreek Methanol extract had presented that it has a high value of phenolic and flavonoid compounds that were 154.68 ± 1.50 μg of GAE/mg of the extract and 37.69 ± 0.73 μg of the QE/mg of the extract, respectively. Also, we noticed that there are flavanoid-C-glycoside compounds, such as vicenin, isoschaftoside, and isoorientin. Although the pathophysiology of diabetes is not fully understood, many studies have reported the role of free radicals in the pathogenesis of diabetes and its complications [102]. We noted through our results that fenugreek seeds have strong antioxidant activity, as indicated by many studies [103]. This is because the main bioactive compounds in fenugreek seeds are polyphenols [104], which may be beneficial for the prevention of diabetes and its complications [97].

### 2.4. ADMET and Drug-Likeness Evaluation

We evaluated the pharmacokinetic (absorption, distribution, metabolism, excretion, and toxicity) and drug-likeness properties of the flavonoids from fenugreek seeds to be accepted as a treatment for diabetes “see Table 4 below”. Compounds C2 and C9 showed good drug-likeness characteristics; however, the rest of the flavonoids violated the Lipinski rule. All compounds were predicted to be non-substrates or non-inhibitors for CYP (CYP1A2, CYP2C9, CYP2D6, CYP2C19, and CYP3A4) except C5, C6, C8, and C10, which they were predicted to be P-gp Substrate. These properties are not particularly significant, as the target is found in the digestive tube. All studied compounds were also predicted to be non-carcinogens, while all of them showed potential hepatotoxicity and mutagenesis with a probability of 0.4 to 0.7, including the FDA-approved drug acarbose.

### 2.5. Molecular Docking

Molecular docking methods are commonly used in medicinal chemistry research for calculating the affinity of molecules toward protein targets and predicting the interactions within complexes [105].

**Figure 2:**
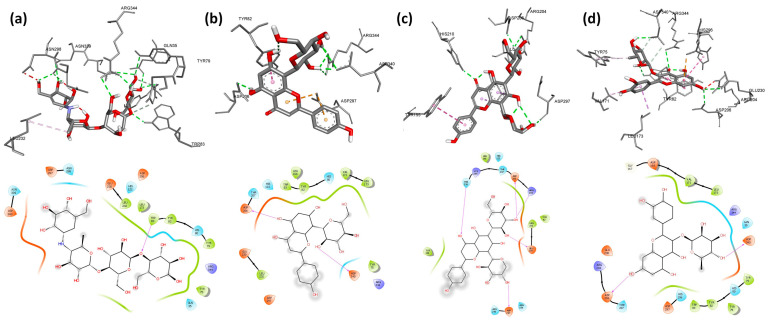
3D and 2D pictures representing the binding modes of the pocket of *A. oryzae* α-amylase (7TAA) with the best-selected flavonoids: (**a**) acarbose, (**b**) vitexin, (**c**) schaftoside, (**d**) quercetin 3-rhamnoside.

**Figure 3:**
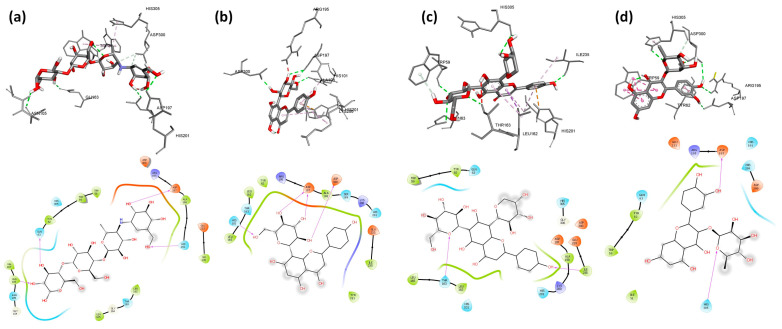
3D and 2D pictures representing the binding modes of the pocket of Human Pancreatic α-amylase (2qv4) with the best-selected flavonoids: (**a**) acarbose, (**b**) vitexin, (**c**) schaftoside, (**d**) quercetin 3-rhamnoside.

**Figure 4:**
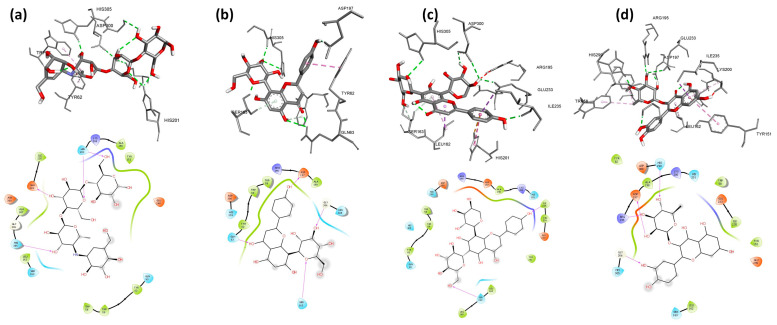
3D and 2D pictures representing the binding modes of the pocket of Human Salivary α-amylase (3DHP) with the best-selected flavonoids: (**a**) acarbose, (**b**) vitexin, (**c**) schaftoside, (**d**) quercetin 3-rhamnoside.

Firstly, we run a validation process to verify the accuracy and precision of our docking program. We validated the behavior of the co-crystallized inhibitor: acarbose for *A. oryzae* α-amylase for human pancreatic α-amylase by a low RMSD value of 0.58 Å and 0.55 Å, respectively. Molecular docking of nine flavonoids from fenugreek seeds (C1–C9) and acarbose (C10) into the α-amylases active site was performed. The results are shown in Table 5, and their binding conformations are represented in Figure 2, Figure 3 and Figure 4. The resultant data were regarded as valid since redocking the crystallized ligands produced good ligand superposition with RMSD less than 2 Å [106].

Depending on the values of the docking score shown in Table 5, the descending order of binding affinity is quercetin 3-rhamnoside > vitexin > orientin > isoorientin > schaftoside > isovitexin and vicenin3 > vicenin1 and vicenin2 > acarbose for *A. oryzae* α-amylase, and schaftoside and vicenin3 > quercetin 3-rhamnoside > orientin > vicenin1 > isovitexin and acarbose > isoorientin and vicenin2 and vitexin for human pancreatic α-amylase. In contrast, for human salivary α-amylase, the descending strength order was schaftoside > quercetin 3-rhamnoside > vicenin3 > vicenin2 and vitexin > isoorientin and orientin and vicenin1 > isovitexin > acarbose.

**Figure 5:**
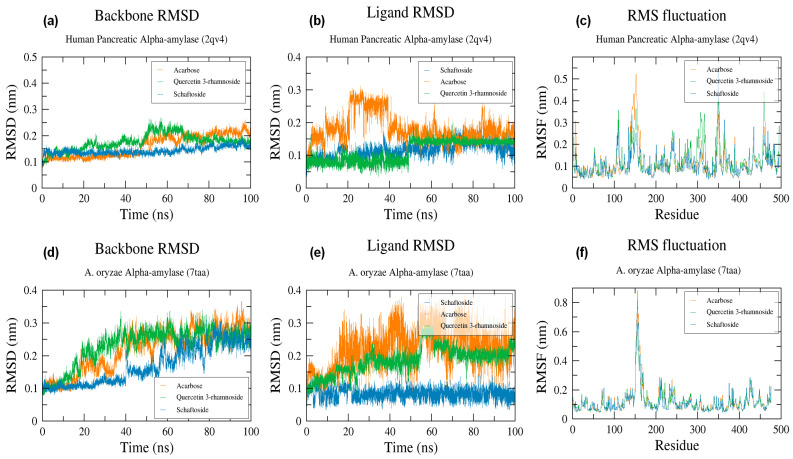
Molecular dynamic simulation results: plots of backbone RMSD, ligand RMSD and RMSF of the studied complexes; (**a**,**d**) RMSD changes in α-amylases complex backbones, (**b**,**e**) RMSD change in ligands, (**c**,**f**) RMSF of α-amylases residues throughout the 100-ns MD simulation.

Based on the results presented in Table 5, the studied flavonoids were very close in binding scores and binding modes to the co-crystallized inhibitor (acarbose), especially schaftoside and quercetin 3-rhamnoside with binding scores of −8.7 and −8.6 Kcal/mol, respectively, towards human pancreatic α-amylase, indicating promising binding affinity with expected effective inhibition activity. As shown in Table 5, the present interaction showed the formation of hydrogen bonds between all nine studied flavonoids and residues in the active site of α-amylases. The following hydrogen bonds (five) were formed between schaftoside and the human pancreatic α-amylase residues: Thr163 (2.22 Å), Ile235 (2.42 Å), Trp59 (2.62 Å) and His305 (2.51; 2.79 Å) (Figure 3c). Schaftoside formed also six hydrogen bonds with human salivary α-amylase as follows: Ser163 (2.67 Å), Ile235 (2.05 Å), His305 (2.81 Å), Asp300 (2.40; 2.84 Å) and Glu233 (2.18 Å) (Figure 5c), and six hydrogen bonds with *A. oryzae* α-amylase: Arg204 (1.95; 2.79 Å), His210 (2.12 Å), Asp206 (2.08 Å), Glu230 (2.05 Å) and Asp297 (2.06 Å) (Figure 2c). It is worth mentioning that in addition to hydrogen bonds, schaftoside is also stabilized by the hydrophobic interactions that form with the amino acids of the active pocket. Furthermore, the interaction of quercetin 3-rhamnoside on α-amylases formed hydrogen bonds as follows: four H-bonds with the human pancreatic α-amylase: His305 (2.38 Å), Asp300 (2.23; 2.89 Å) and Asp197 (2.13 Å); five H-bonds with the human salivary α-amylase: Arg195 (2.58 Å), His299 (1.81 Å), Gly306 (2.13 Å), Asp197 (1.96 Å) and Glu233 (2.93 Å); five H-bonds with *A. oryzae* α-amylase: His80 (2.58 Å), Arg344 (3.20 Å), Asp340 (2.21 Å), Asp206 (2.46 Å) and Glu230 (2.62 Å). These hydrogen bonds are supported by more than six hydrophobic interactions. In general, both schaftoside and quercetin 3-rhamnoside were stabilized at the binding site by multiple and diverse interactions (Table 5). In comparison with the reference molecule acarbose, the current approved α-amylase inhibition drug, where the acarbose forms only three hydrophobic interactions and six hydrogen bonds with Ala106 (1.95 Å), His201 (2.52; 2.68 Å), Asp197 (2.50 Å), Asn105 (2.44 Å), and Gln63 (2.09 Å) of human pancreatic α-amylase. The binding mode and the formed interactions with the amino acids of the binding pocket of the best-selected flavonoids and acarbose are represented in Figure 2, Figure 3 and Figure 4.

According to these observations, we suggest that schaftoside and quercetin 3-rhamnoside are good inhibitors for α-amylase and could be promising molecules to develop new drugs against diabetes. It is worth mentioning that the other flavonoids have also presented a good binding score and binding mode similar to acarbose, where they formed a significant number of hydrogen bonds and hydrophobic interactions with amino acids of the active site.

### 2.6. Molecular Dynamics Simulation

**Figure 6:**
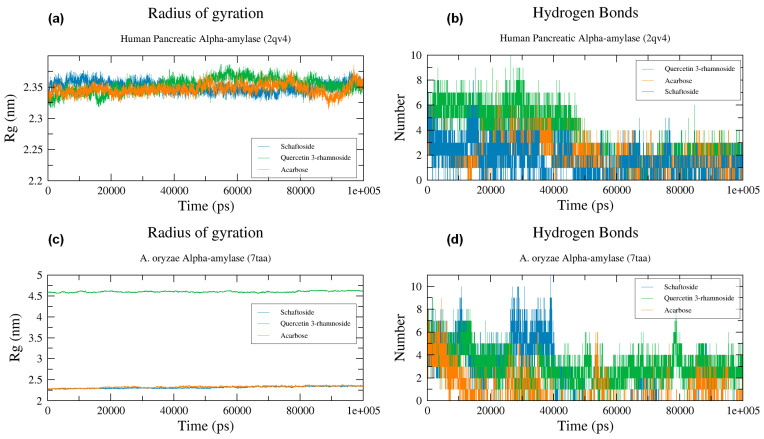
The radius of gyration and the number of hydrogen bonds of the studied complexes. (**a**,**c**): Total Rg change over time, (**b**,**d**) Number of hydrogen bonds during the 100-ns MD.

The molecular dynamic simulation was performed to examine the structural stability of the protein complexes and estimate the different bonds formed between the protein target and small molecules during 100 ns MD simulation. MD simulation revealed that the stable structures were preserved, as shown in Figure 5 and Figure 6. The human pancreatic α-amylase in complex with schaftoside was the most stable with RMSD less than 2 Å.

#### 2.6.1. RMSD

Schaftoside and quercetin 3-rhamnoside exhibited a stable binding to human pancreatic α-amylase, as confirmed by minor changes in RMSD of the ligand (up to 1.5 Å) and protein backbone (up to 2 Å and 2.5 Å for schaftoside and quercetin 3-rhamnoside respectively), while RMSD of acarbose showed a big deviation between 10 and 40 ns (Figure 5b). For *A. oryzae* α-amylase, RMSD of the backbone increased gradually in the first 60 ns, then remained stable around 2.8 Å till the end of the simulation in the three cases (schaftoside, quercetin 3-rhamnoside, and acarbose) (Figure 5d). The backbone RMSD plot suggests that schaftoside and quercetin 3-rhamnoside show the same affinity towards human pancreatic α-amylase and do not disturb the structural stability of the enzyme.

#### 2.6.2. RMSF

RMSF analysis of human pancreatic α-amylase showed low fluctuation around 3 Å except for residues 152, 349, and 350, which showed a notable fluctuation reaching 6 Å, respectively (Figure 5c). RMSF analysis of *A. oryzae* α-amylase also showed a small fluctuation of less than 2 Å except for residues 156–162, which showed a notable fluctuation that reached 8 Å respectively (Figure 5f). It has been confirmed that the loops can often present conformational changes [107,108].

#### 2.6.3. Radius of Gyration

The radius of gyration (Rg) measures the compactness of a protein and shows the effect of the presence of a ligand on the protein structure [109]. The constant values of Rg show that the ligand does not affect the protein structure, while the big fluctuations indicate the instability of protein folding and change of protein structure [110,111]. The Rg plots of all studied complexes of both *A. oryzae* α-amylase and human pancreatic α-amylase were stable throughout 100 ns MD simulation (Figure 6a,b). These results indicated that the structural stability did not deteriorate during the MD simulation, and the enzymes preserved their native structure and were not affected by the ligands during the simulation.

#### 2.6.4. Hydrogen Bonds

The binding affinity of schaftoside, quercetin 3-rhamnoside, and acarbose towards both types of α-amylases was confirmed by checking the intermolecular hydrogen bonds, where the formation of hydrogen bonds in the complex leads to strong binding [112].

MD simulation revealed that schaftoside, quercetin 3-rhamnoside, and acarbose interacted strongly with human pancreatic α-amylase, where they formed a minimum of 3 hydrogen bonds with active site residues during 100 ns MD simulation (Figure 6b). Whereas *A. oryzae* α-amylase schaftoside, quercetin 3-rhamnoside, and acarbose also formed several hydrogen bonds around six hydrogen bonds at the beginning of the simulation and about 3 HBs at the end of the simulation (Figure 6d). However, the stability of the studied molecules over time can be explained by the hydrogen bonds formed in the complexes alongside the high number of hydrophobic interactions formed between the ligands and α-amylases.

### 2.7. Principal Component Analysis (PCA)

**Figure 7:**
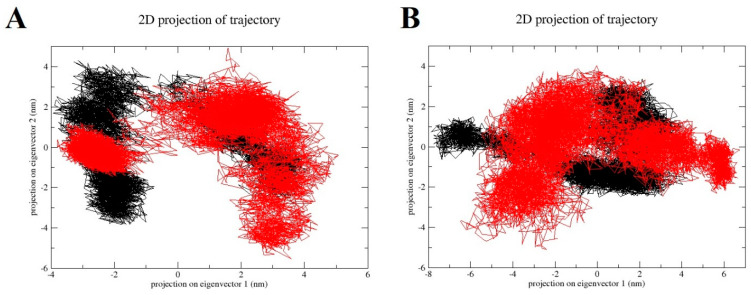
(**A**) PCA plots show the projections of the displacement of the backbone of 2QV4 in complexed with QU (black) and of 2QV4 in complexed with ac (red), and (**B**) PCA plots show the projections of the displacement of the backbone of 7TAA in complexed with QU (black) and of 7TAA in complexed with AC (red) 7TAA.

**Figure 8:**
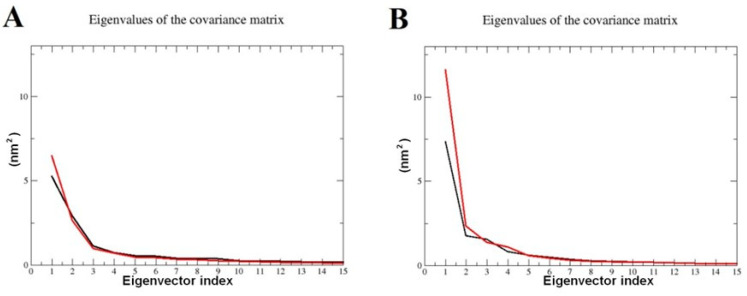
The cumulative contribution of the 14 most relevant eigenvectors to the variance of the overall motion of (**A**) 2QV4 complexed with QU (black) and 2QV4 complexed with AC (red), (**B**) 7TAA in complexed with QU (black) and of 7TAA in complexed with AC (red) 7TAA.

PCA analyses were performed on the MD trajectories of 2QV4 with the QU and AC ligands. The total motions of backbone atoms were dispersed over 4456 eigenvectors. The combined contributions of the eigenvectors with the top two eigenvalues were 44% and 52% for the 2QV4 molecule with the QU and AC, respectively. PC1 and PC2 were plotted together to visualize the essential sub-space of collective motions (Figure 7). The eigenvalues taken from the diagonalization of the covariance matrix of the fluctuation of backbone atoms. The overall flexibility of both QU–2QV4 complex and AC–2QV4 complex was seen by the trace of the covariance matrix offer diagonalizing obtained for QU–2QV4 is 18.7302 nm2 and for AC–2QV4 17.6181 nm2. It can be observed that the range of the PC1 values is a little larger for the 2QV4–QU complex compared to other AC–2QV4 complexes (Figure 7A), indicating that the 2QV4–QU is relatively more flexible with respect to the motion depicted by eigenvector 1 (Figure 8A). For QU–7TAA and AC–7TAA complexes, PCA analysis was performed, and combined contributions of the eigenvectors with the top two eigenvalues were 49% and 59%, respectively. The plots of PC1 against PC2 of QU–7TAA and AC–7TAA are presented in Figure 7B. The total flexibility for both QU–7TAA complex and AC–7TAA complex was seen by the trace of the covariance matrix offer diagonalizing were 18.4854 nm2 and for AC–2QV4 23.3184 nm2.

#### Free-Energy Landscape

**Figure 9:**
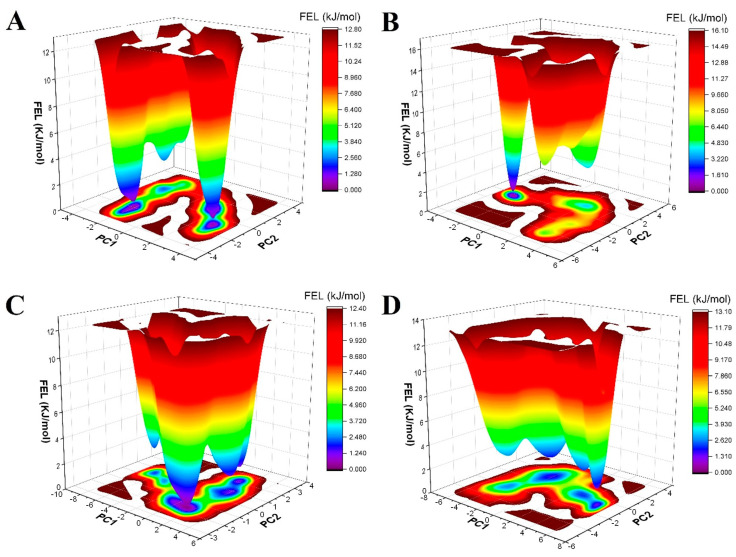
Gibbs free energy landscape calculated from PC1 and PC2 of (**A**) 2QV4–QU complex, (**B**) of 2QV4–AC complex, (**C**) 7TAA–QU complex, and (**D**) of 7TAA–AC complex.

Information on FEL is used to determine the most energetically favorable structure [113]. The minima of global energy states are shown in violet. Figure 9A shows the FEL of (A) 2QV4–QU complex and (B). As can be seen, the 2QV4–AC complex displayed a single dominant minimum energy basin observed. 2QV4–QU (Figure 9B) contains four clear single points of the lowest Gibbs energy, but with the single lowest energy that is much broader than the basins of 2QV4–AC. When QU and AC bound to 7TAA, FEL showed three distinct minima regions for QU (Figure 9C), and FEL also showed three distinct minima regions for AC (Figure 9D).

## 3. Materials and Methods

### 3.1. Sample Preparation

#### 3.1.1. Plant Material

The fenugreek (*Trigonella foenum-graecum* L.) seeds were harvested in the Bin El Ouiden region in the spring of 2012 (Skikda Province, northeastern Algeria), cleaned, and air-dried. Ten g of seeds were ground into a fine powder using an electric grinder and then stored at 4 °C until analysis.

#### 3.1.2. Chemicals: Reagents and Standards

LC-MS-grade acetonitrile and methanol were purchased from Merck (Darmstadt, Germany), and trifluoroacetic acid (TFA, 99% purity) from Sigma-Aldrich (Darmstadt, Germany). All other chemicals and solvents used were of analytical grade. The standards of flavonoids: vicenin-1, vicenin-2, vicenin-3, schaftoside-1, schaftoside-2, isoorientin, orientin, isovitexin, vitexin, and quercetin 3-O-rhamnoside originated from the set of standards of the Department of Pharmacognosy of the Medical University of Gdańsk (Poland). Water was from a Millipore system (Merck, Darmstadt, Germany).

Butylated hydroxyanisole (BHA), hydroxytoluene (BHT), α-tocopherol, 1,1-diphenyl-2-picrylhydrazyl (DPPH●), 2,2′-azinobis(3-ethylbenzothiazoline-6-sulfonic acid) diammonium salt (ABTS●+), trichloroacetic acid (TCA), potassium ferricyanide, neocuproine, sodium phosphate monobasic dihydrate and α-amylase were purchased from Sigma Chemical Co. (Sigma-Aldrich GmbH, Sternheim, Germany). Folin–Ciocalteu reagent and gallic acid were purchased from Panreac (Barcelona, Spain). Ascorbic acid was obtained from Sigma (St. Louis, MO, USA). Aluminum nitrate (Al(NO_3_)_3_, 9H_2_O), iron (III) chloride (FeCl_3_), copper (II) chloride (CuCl_2_), potassium persulfate (K_2_S_2_O_8_), potassium acetate (CH_3_CO_2_K) and ammonium acetate which were obtained from Biochem Chemopharma (France). Acarbose was purchased from Fluka (Bucharest, Romania). Potato starch was obtained from Fisher (Pittsburgh, PA, USA).

#### 3.1.3. Plant Extract Preparation

The extraction of fenugreek seeds was performed using the Soxhlet apparatus; ten grams (10 g) of powdered fenugreek seeds were placed in a Soxhlet apparatus and extracted thoroughly in a Soxhlet apparatus in three steps. The plant material was first defatted with hexane, then chloroform, and finally methanol. The obtained methanol extract was filtered and concentrated to dryness using a rotary evaporator. Then, the extraction yield was evaluated according to [114] as follows:

(1) $$Extraction\;yield\%=\frac{Weight\;of\;extracts\;from\;plant\;sample}{Weight\;of\;dried\;plant\;sample}\times 100$$

### 3.2. Phytochemical Composition

#### 3.2.1. Determination of Total Phenolic Contents (TPC)

The total phenolic content was determined using Folin–Ciocalteu reagent according to the method described by [115]. As follows: 20 μL of the sample was mixed with 100 μL of Folin–Ciocalteu reagent (10%), and 75 μL of aqueous sodium carbonate solution (7.5%, *w*/*v*), then the mixture was incubated at room temperature and in the dark for 2 h. The absorbance was measured at 765 nm using a microplate reader of type PerkinElmer (Enspire). Gallic acid was used as a standard for plotting the calibration curve. The total phenolic content was expressed as milligrams of gallic acid equivalents (GAE) per gram of dry weight.

#### 3.2.2. Determination of Flavonoid Content (FC)

The total flavonoid content was determined according to the [116] method. Quercetin was used as a standard to plot the calibration curve. 50 μL of sample or standard was incubated with 130 μL of methanol, 10 μL of potassium acetate (9.8%, *w*/*v*), and 10 μL aluminum nitrate nonahydrate (Al (NO_3_)_2_, 9H_2_O) (10%, *w*/*v*), in room temperature and dark for 40 min. After incubation, the absorbance was measured at 415 nm using a microplate reader of type PerkinElmer (Enspire). The flavonoid content was expressed as milligrams of quercetin equivalents (QE) per g dry weight.

#### 3.2.3. HPLC-DAD-ESIMS analysis

HPLC-DAD-ESI-MS system (Shimadzu, Japan) was used. Separations were performed on a Kinetex PFP column (100 × 46 mm, 2.6 µm) (Phenomenex, Torrance, CA, USA) with the gradient elution program: 0 min—10% B, 12 min—16% B, 25 min—30% B, 30 min—50%, 35 min—100% B, A: water/trifluoroacetic acid, 100:0.1 (*v*/*v*), B: acetonitrile/trifluoroacetic acid, 100:0.1 (*v*/*v*), at column temp. 25 °C, flow rate 1.0 mL/min, detection UV at λ—330 nm. Mass spectra were acquired in positive (PI) and negative (NI) ion modes, the nebulizing gas (nitrogen) flow was 1.5 L/min, the desolvation line and block temperature were 250 °C and 200 °C, respectively, interface voltage was 4.5 kV, the detector voltage was 2.5 kV [E−] and 3.0 kV [E+] and the drying gas (nitrogen) flow was 10 L/min. The SIM technique was used for monitoring the specific signals of selected ions.

### 3.3. Anti-α-amylase Assay

The inhibition assay of methanolic extracts of Fenugreek seeds was carried out using the iodine/potassium iodide (IKI) method [117] with some modifications, as follows:

25 μL of extracts at different concentrations were added to 50 μL of *A. oryzae* α-amylase enzyme (1U), and the mixture was preincubated for 10 min at 37 °C. Then, the reaction was initiated by adding 50 μL of starch (0.1%). After 10 min of incubation at 37 °C, the reaction was stopped by adding 25 µL of HCl (1M) and 100 µL of IKI, and then the absorbance was measured at 630 nm using a microplate reader of type PerkinElmer (Enspire). The inhibition of α-amylase was calculated using the following equation:

(2) $$Inhibition\left(\%\right)=1-\left[\frac{\left(Ac-Ae\right)-\left(As-Ab\right)}{\left(Ac-Ae\right)}\right]$$

where:

Ac = Absorbance [25 μL Solvent + 50 μL buffer solution + starch + HCl + IKI];

Ae = Absorbance [25 μL Solvent + enzyme + starch + HCl + IKI];

As = Absorbance [Extract + enzyme + starch + HCl +I KI];

Ab = Absorbance [Extract + 125 µL buffer solution + IKI].

The FDA-approved drug acarbose was used as a reference. The inhibitory activity of the extracts and standards was expressed as IC_50_ (the concentration of the tested samples that inhibits 50% of α-amylase).

### 3.4. Antioxidant Tests

#### 3.4.1. DPPH Assay

The ability of radical scavenging was determined according to [118], using the radical DPPH• (2,2-diphenyl-1-picrylhydrazyl). 160 µL of DPPH solution (0.15 mM) was added to 40 µL of the diluted extracts in methanol and incubated in the dark. After 30 min of incubation, the absorbances were measured at 517 nm by a microplate reader of type PerkinElmer (Enspire) against a blank. BHA, BHT, and α-tocopherol were used as reference antioxidants. DPPH inhibition percentage was calculated using the following equation:

(3) $$Inhibition(\%)=\frac{\left(A0-As\right)}{A0}\times 100$$

where A0 is the absorbance of the control (without the sample) and As is the absorbance of the tested samples. The antioxidant activity of the extracts and standards was expressed as IC_50_ (the concentration of the tested samples that inhibits 50% of DPPH radicals).

#### 3.4.2. Ferric-Reducing Antioxidant Power Assay

The ferric-reducing antioxidant power (FRAP) assay was carried out according to [119]. The reaction mixture contained 10 μL of the extracts, 40 µL phosphate buffer (pH 6.6), and 50 μL potassium ferricyanide K_3_[Fe (CN)_6_] (1%), was incubated at 50 °C for 20 min, then 50 µL of trichloroacetic acid (TCA) (10%) and 40 µL of distilled water and 10 µL of ferric chloride FeCl_3_ (0.1%) were added. Then, the absorbance was measured at 700 nm using a microplate reader of type PerkinElmer (Enspire) against a blank. Tannic acid, α-tocopherol, and ascorbic acid were used as references.

#### 3.4.3. ABTS+ Scavenging Activity

The ABTS•+ assay (2, 2′-azino-bis (3-ethylbenzthiazoline-6-sulfonic acid)) was carried out according to the [120] method with some modifications. The ABTS+ reagent was prepared as follows: 19.2 mg of ABTS (7 mM) in 5 mL distilled water was mixed with 3.3 mg of Potassium persulfate K_2_S_2_O_8_ (2.45 mM) in 5 mL distilled water, and the mixture was incubated for 16 h in the dark before use. We have added 160 µL of ABTS+ reagent to 40 μL of extract solutions. After 10 min of incubation, the absorbance was read at 734 nm using a microplate reader of type PerkinElmer (Enspire). BHA and BHT were used as references. The total antioxidant activity of the extracts was calculated as follows:

(4) $${ABTS}^{+}scavenging\;effect\;(\%)=\frac{\left(A_{Control}-A_{Sample}\right)}{\begin{array}{c} A_{Control} \end{array}}\times 100$$

#### 3.4.4. Cupric Reducing Antioxidant Capacity (CUPRAC) Assay

Cupric reducing antioxidant capacity was determined using the [121] method. 40 µL of the extracts were mixed with 60 µL of ammonium acetate (NH_4_CH_3_CO_2_), and 50 µL of copper (II) chloride dihydrate (CuCl_2_, 2H_2_O), then 50 µL of neocuproine was added. After 1 h of incubation, the absorbance was read at 450 nm using a microplate reader of type PerkinElmer (Enspire). BHA and BHT were used as references.

### 3.5. ADMET and Drug-Likeness Evaluation

**Figure 10:**
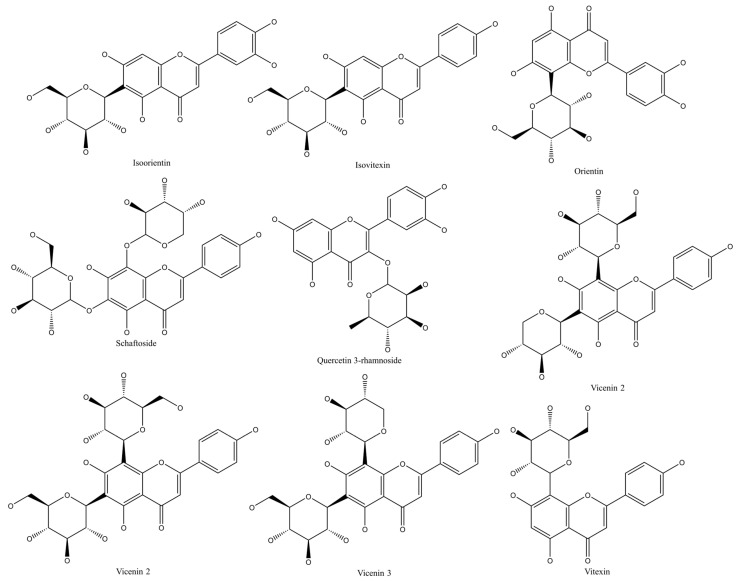
Structure of flavonoids detected in methanolic extract of fenugreek seeds.

ADME/Tox evaluation is an important process for selecting a good drug candidate [122]. The drug-likeness and toxicity of any compound can be evaluated using webservers that calculate some properties of a molecule based on its structure. In this study, we used two web servers: The swissADME server (http://www.swissadme.ch/index.php [123] (accessed on 15 January 2023)) and the admetSAR 2.0 server http://lmmd.ecust.edu.cn/admetsar2) [124] (accessed on 15 January 2023). We obtained The SMILES codes of the studied compounds (Figure 10) from the PubChem Database [125].

### 3.6. Molecular Docking

We have performed a specific molecular docking simulation to study the interactions between the compounds (Figure 10) from fenugreek seeds and α-amylase enzymes. The enzyme α-amylase is considered the main target for the development of anti-diabetic drugs. We have prepared the input files using Autodock tools (ADT) (version 1.5.4) [126] as follows: We have downloaded the crystal structure of α-amylases (PDB IDs: 3DHP, 2QV4, and 7TAA) from Protein Data Bank (PDB) [127]. The files were assembled, and then we removed the water molecules, co-crystallized solvent, ligands, and other heteroatoms. We have added polar hydrogens and partial charges, and the docking box was set based on the co-crystallized inhibitor. The size of the box and centers are presented in Table 6. Finally, we have run the molecular docking using Autodock Vina 1.1.2 software. We have set 50 runs with one conformation for each compound. After the docking finish, we loaded the output files into the Discovery Studio visualizer (4.0). The most repeated conformation with less binding energy was considered the most stable and chosen for the analysis [128,129,130].

### 3.7. Molecular Dynamics Simulation

After the molecular docking, we checked the stability of the selected complex’s protein–ligand (2QV4–acarbose, 2QV4–Schaftoside, 2QV4–QuercetinO-rhamnoside, 7TAA–acarbose, 7TAA–Schaftoside, and 7TAA–QuercetinO-rhamnoside) by molecular dynamics simulation using Gromacs 2022 [131] according to the method described in [105]. Firstly, we generated the ligands topology files using the CGenFF webserver (https://cgenff.umaryland.edu) (accessed on 15 June 2022) [132] and target topology files using charmm-gui. Then, we put the complex in a rectangular box that contains water molecules (TPT3) and neutralized the system by adding Na+ ions. After the energy minimization, we heated the system to 300 K for 1000 picoseconds. The temperature and the pressure remained constant at around 300 K and 1 atm, respectively [133]. Finally, we run the MD simulation for 100 ns. The recording was made for every 10 ps [134]. After the simulation was finished, we analyzed the trajectory files using Gromacs build-in tools to study the dynamic conformational changes and the interaction in the complexes throughout time. We have used XMGRACE to plot the graphs [135].

### 3.8. Statistical Analysis

The results of the tests carried out are expressed as an average ± SD of analyses in three tests. The values of IC_50_ (50% inhibition concentration) and A0.5 (the concentration indicating 0.50 absorbance) are calculated using the linear regression method from the two curves: [% inhibition = f (concentration)] for IC_50_ and [Absorbance = f (concentration)] for A0.5. All the antioxidant and enzymatic tests were carried out at more than four concentration values. The one-way analysis of variance ANOVA was used to detect significant differences (*p* < 0.05).

## 4. Conclusions

Diabetes is a chronic metabolic disorder characterized by high blood glucose levels resulting from a deficiency in insulin secretion or its inability to function effectively. One promising approach to managing diabetes is through the use of natural plant-based remedies that can regulate blood sugar levels by inhibiting enzymes such as amylase, which breaks down carbohydrates into glucose. In this work, the methanolic fraction was extracted using the Soxhlet extraction technique, and the phytochemical profile, antioxidant, and α-amylase inhibitory obtained from the seeds of *Trigonella foenum-graecum* have been evaluated by using an HPLC-DAD-ESI-MS analysis, twenty-seven flavonoid compounds were identified, including twenty-two flavones and three flavonols. Higher levels of phenolic compounds in fenugreek showed stronger antioxidant activities and inhibitory potency of amylase enzyme. The major flavonoid compounds that were found in the methanolic extract of fenugreek seeds showed good amylase enzyme inhibitory activities. Molecular docking analysis revealed the underlying inhibition mechanisms of these compounds against amylase enzyme; the studied flavonoids were also very close in binding scores and binding patterns of acarbose towards human α-amylase, indicating a promising binding affinity with expected potent inhibition activity. The findings of this study would urge more research into high-degree isolation, structural re-determination, and in-depth bio-assaying of the most promising compounds.

## Figures and Tables

**Table 1:** Chromatographic and spectral data of C-glycosylflavones identified by HPLC-DAD-ESI-MS in the fenugreek seeds.

Peak no.	tR min	UV [λ_max_ nm]	ESI Scan (+) *m*/*z* 250–1200	ESI Scan (−) *m*/*z* 250–1300	Compound
1	5.827	270, 334	595	593, 707 (TFA)	Vicenin-2
2	7.896	269, 333	565	563, 677 (TFA)	Vicenin-1
3	8.698	269, 333	565	563, 677 (TFA)	Schaftosid-2
4	8.763	270, 333	565	563, 677 (TFA)	Schaftoside-1
5	9.014	257, 268 (s), 347	449	447, 561 (TFA)	Isoorientin
6	9.504	268, 324	949	947, 1061 (TFA)	KaempferolO-feruloyl-triglucoside/trigalactoside isomer
7	9.779	271, 332	565	563, 677 (TFA)	Apigenin 6,8-C-pentoside-hexoside isomer
8	10.138	255, 266 (s), 347	449	447, 561 (TFA)	Orientin
9	10.554	270, 334	565	563, 677 (TFA)	Vicenin-3
10	11.890	270, 335	535	533, 647 (TFA)	Apigenin-C-di-(6/8)-pentoside isomer
11	11.890	270, 335	433	431, 545 (TFA)	Isovitexin
12	12.187	267, 335	433	431, 545 (TFA)	Vitexin
13	12.594	253, 265, 315, 349 (s)	935	933, 1047 (TFA)	Quercetin O-coumaroyl-tri-glucoside/trigalactoside isomer
14	12.816	270, 335	535	533, 647 (TFA)	Apigenin-C-di-(6/8)-pentoside isomer
15	13.130	270, 335	535	533, 647 (TFA)	Apigenin-C-di-(6/8)-pentoside isomer
16	13.648	270, 334	535	533, 647 (TFA)	Apigenin-C-di-(6/8)-pentoside isomer
17	14.434	270, 339	535	533, 647 (TFA)	Apigenin-C-di-(6/8)-pentoside isomer
18	15.016	269, 331	535	533, 647 (TFA)	Apigenin-C-di-(6/8)-pentoside isomer
19	16.024	269, 330	549	547, 661 (TFA)	Apigenin C-di-(6,8)-pentoside methyl ether
20	16.976	267, 315	-	-	Probably Unknown flavonoid ester
21	17.181	250, 263, 327 (s), 361	449	-	Quercetin 3-O-rhamnoside
22	18.115	243, 314	-	647, 761 (TFA), 681, 795 (TFA)	Probably Unknown flavonoid ester
23	18.730	274, 339	-	-	Unknown flavone
24	19.170	310	-	-	Unknown
25	20.303	274, 329	-	707, 875	Unknown flavone
26	21.425	270, 316	595	593, 707 (TFA)	p-coumaroyl-orientin/isoorientin isomer
27	22.435	270, 315	579	577, 691 (TFA)	p-coumaroyl vitexin/isovitexin isomer
28	23.211	270, 315	579	577, 691 (TFA)	p-coumaroyl vitexin/isovitexin isomer

Abbreviations: tR, retention time; (s) shoulder.

**Table 2:** The extraction yield, total phenolic content, flavonoid content, and ani- α-amylase of the methanolic extract from fenugreek seeds.

	Yield %	TPC (µgGAE/mg Extract)	TFC (µgQE/mg Extract)	α-Amylase Inhibition IC_50_ (µg/mL)
Fenugreek seeds	17.6	154.68 ± 1.50	37.69 ± 0.73	653.52 ± 3.24
Acarbose	-	-	-	3650.93 ± 10.70

**Table 3:** The antioxidant activities of the methanolic extract from fenugreek seeds.

	DPPH Inhibition IC_50_ (µg/mL)	FRAP A0.5 (µg/mL)	ABTS Inhibition IC_50_ (µg/mL)	CUPRAC A0.5 (µg/mL)
Fenugreek seeds	556.6 ±9.87	>200	593.62 ± 9.35	451.90 ± 9.07
BHA	6.14 ± 0.41	n.d	1.81 ± 0.10	-
BHT	12.99 ± 0.41	n.d	1.29 ± 0.30	-
Tannic acid	n.d	5.39 ± 0.91	n.d	n.d
Ascorbic acid	n.d	6.77 ± 1.15	n.d	n.d

n.d: not determined.

**Table 4:** ADMET and drug-likeness of acarbose (C10) and nine flavonoids (C1–C9) from fenugreek seeds.

	Molecule	C1	C2	C3	C4	C5	C6	C7	C8	C9	C10
Drug - likeness	Lipinski	-	+	-	-	-	-	-	-	+	-
	Blood-Brain Barrier	-	-	-	-	-	-	-	-	-	-
Absorption	Human Intestinal Absorption	Low	Low	Low	Low	Low	Low	Low	Low	Low	Low
	Caco-2	-	-	-	-	-	-	-	-	-	-
	Human oral bioavailability	-	-	-	-	-	-	-	-	-	-
	Skin Permeation	−9.14 cm/s	−8.79 cm/s	−9.14 cm/s	−8.42 cm/s	−11.30 cm/s	−11.30 cm/s	−11.53 cm/s	−11.30 cm/s	−8.79 cm/s	−16.29 cm/s
Metabolism	P-glycoprotein Inhibitor	-	-	-	-	-	-	-	-	-	-
	P-gp Substrate	-	-	-	-	+	+	-	+	-	+
	CYP450 1A2 Inhibitor	-	-	-	-	-	-	-	-	-	-
	CYP450 2C9 Inhibitor	-	-	-	-	-	-	-	-	-	-
	CYP450 2D6 Inhibitor	-	-	-	-	-	-	-	-	-	-
	CYP450 2C19 Inhibitor	-	-	-	-	-	-	-	-	-	-
	CYP450 3A4 Inhibitor	-	-	-	-	-	-	-	-	-	-
Toxicity	AMES mutagenesis (probability)	+(0.610)	−(0.550)	+(0.710)	+(0.770)	+(0.510)	+(0.510)	+(0.600)	+(0.510)	+(0.540)	+(0.520)
	Carcinogens (probability)	−(0.986)	−(0.986)	−(0.986)	−(0.986)	−(0.986)	−(0.986)	−(0.986)	−(0.986)	−(0.986)	−(1)
	Hepatotoxicity (probability)	+(0.600)	+(0.600)	+(0.600)	+(0.650)	+(0.675)	+(0.675)	+(0.650)	+(0.675)	+(0.675)	−(0.625)

**Table 5:** The molecular docking results: Repeating ratio of the chosen conformation, binding scores, and amino acid interactions of nine flavonoids from fenugreek seeds (C1–C9) and acarbose (C10) in the binding pocket of three types of α-amylases (*A. oryzae*, human pancreatic, and human Salivary).

	*A. oryzae*					Human Pancreatic					Human Salivary				
Ligand	RR%	Affinity (Kcal/mol)	H-I	NHB	Hydrogen Bonds	RR%	Affinity	H-I	NHB	Hydrogen Bonds	RR%	Affinity	H-I	NHB	Hydrogen Bonds
C1: Isoorientin	100	−8.2	6	4	Glu230 (1.90; 2.85 Å) Asp206 (2.64 Å) Asp233 (1.98 Å)	58	−8.2	4	3	Arg195 (2.10 Å) Glu233 (2.93 Å) Asp300 (2.44 Å)	100	−8.7	7	4	Ser163 (2.80; 2.01 Å) Lys200 (2.90 Å) Glu233 (2.79 Å)
C2: Isovitexin	100	−8.0	6	5	Glu230 (2.04; 2.12; 2.92 Å) Asp206 (2.99 Å) Glu156 (2.86 Å)	92	−8.3	4	3	Tyr62 (2.30 Å) Thr163 (2.21; 2.28 Å)	54	−8.6	6	2	Ile235 (1.78 Å) His305 (2.62 Å)
C3: Orientin	100	−8.4	3	4	Trp83 (2.47 Å) Arg344 (2.92 Å) Asp340 (2.91; 3.05 Å)	100	−8.5	5	3	Tyr62 (2.62 Å) Asp197 (2.08 Å) Glu233 (2.16 Å)	100	−8.7	1	5	Ser163 (2.40 Å) Arg195 (2.67 Å) His305 (2.22 Å) Glu233 (2.43; 2.30 Å)
C4: Quercetin 3-rhamnoside	92	−8.8	8	4	His80 (2.58 Å) Asp340 (2.21 Å) Asp206 (2.46 Å) Glu230 (2.62 Å)	100	−8.6	6	4	His305 (2.38 Å) Asp300 (2.23; 2.89 Å) Asp197 (2.13 Å)	100	−9.8	7	5	Arg195 (2.58 Å) His299 (1.81 Å) Gly306 (2.13 Å) Asp197 (1.96 Å) Glu233 (2.93 Å)
C5: Schaftoside	100	−8.1	3	6	Arg204 (1.95; 2.79 Å) His210 (2.12 Å) Asp206 (2.08 Å) Glu230 (2.05 Å) Asp297 (2.06 Å)	72	−8.7	6	5	Thr163 (2.22 Å) Ile235 (2.42 Å) Trp59 (2.62 Å) His305 (2.51; 2.79 Å)	100	−9.9	5	6	Ser163 (2.67 Å) Ile235 (2.05 Å) His305 (2.81 Å) Asp300 (2.40; 2.84 Å) Glu233 (2.18 Å)
C6: Vicenin1	100	−7.8	3	1	His210 (2.21 Å)	72	−8.4	6	5	His101 (2.76 Å) Arg195 (2.51 Å) Lys200 (1.97 Å) Asp197 (2.12 Å) Glu233 (1.64 Å)	98	−8.7	2	6	Gln63 (2.44; 2.71 Å) Ser163 (2.03 Å) His305 (1.78 Å) Asp197 (1.97 Å) Glu233 (2.92 Å)
C7: vicenin2	100	−7.8	3	3	His210 (2.23 Å) Asp206 (2.93 Å) Glu230 (1.95 Å)	98	−8.2	5	8	Tyr151 (2.87 Å) Thr163 (2.22; 2.98 Å) Asp197 (1.74; 2.24 Å) Glu233 (2.57 Å) Asp300 (2.27; 2.71 Å)	62	−8.8	2	5	Ser163 (2.29 Å) Arg195 (2.47 Å) His305 (2.51 Å) Gly306 (2.63 Å) Glu233 (2.20 Å)
C8: Vicenin3	100	−8.0	3	2	His210 (2.19 Å) Asp206 (2.63 Å)	100	−8.7	6	3	Thr163 (2.23 Å) Ile235 (2.40 Å) Trp59 (2.03 Å)	92	−9.0	2	4	Gln63 (2.46 Å) Ser163 (1.94 Å) Asp197 (1.98 Å) Glu233 (2.90 Å)
C9: Vitexin	100	−8.5	3	6	Asp340 (1.97; 2.95 Å) Arg344 (1.95; 2.68; 2.89 Å) Asp206 (2.12 Å)	98	−8.2	5	4	His101 (2.44 Å) Asp300 (1.89 Å) Asp197 (1.97; 2.61 Å)	100	−8.8	1	6	Gln63 (1.97; 2.96 Å) Ser163 (1.92 Å) His305 (2.70 Å) Gly306 (2.11 Å) Asp197 (1.74 Å)
C10: Acarbose	66	−7.5	1	10	Trp83 (2.19 Å) Asn339 (2.85 Å) Arg344 (2.34; 2.87 Å) Asp297 (2.24; 2.42 Å) Gln35 (2.92 Å) Tyr75 (2.47 Å) Tyr79 (2.92 Å) Asp340 (2.83 Å)	100	−8.3	3	6	Ala106 (1.95 Å) His201 (2.52; 2.68 Å) Asp197 (2.50 Å) Asn105 (2.44 Å) Gln63 (2.09 Å)	98	−8.1	2	6	His201 (2.77; 2.05 Å) His305 (2.06 Å) Glu233 (2.65 Å) Asp300 (2.71 Å) Gly306 (2.68 Å)

RR%: repeating ratio; H-I: Hydrophobic interactions; NHB: Number of hydrogen bonds.

**Table 6:** Molecular docking parameters.

Enzyme	PDB ID	Resolution	Co-Crystalized Ligand	Grid Box Center	Grid Box
Human salivary α-amylase	3DHP	1.50 Å	Hydrolyzed substrate	22*20*20	9.103*46.640*19.324
Human pancreatic α-amylase	2QV4	1.97 Å	Acarbose	24*24*24	10.592*47.985*21.039
*Aspergillus oryzae* α-amylase	7TAA	1.98 Å	Acarbose	34*28*22	37.401*41.469*26.378

## Data Availability

All the data in the article are available from the corresponding author upon reasonable request.
